# A meta-analysis of mesophyll conductance to CO_2_ in relation to major abiotic stresses in poplar species

**DOI:** 10.1093/jxb/erab127

**Published:** 2021-03-19

**Authors:** Raed Elferjani, Lahcen Benomar, Mina Momayyezi, Roberto Tognetti, Ülo Niinemets, Raju Y Soolanayakanahally, Guillaume Théroux-Rancourt, Tiina Tosens, Francesco Ripullone, Simon Bilodeau-Gauthier, Mohammed S Lamhamedi, Carlo Calfapietra, Mebarek Lamara

**Affiliations:** 1 Quebec Network for Reforestation and Intensive Silviculture, TELUQ University, Montreal, QC, H2S 3L5, Canada; 2 Forest Research Institute, University of Quebec in Abitibi-Temiscamingue, Rouyn-Noranda, QC, J9X 5E4, Canada; 3 Department of Viticulture and Enology, University of California, Davis, CA 95616, USA; 4 Università degli Studi del Molise, Via De Sanctis, 86100 Campobasso, Italy; 5 Estonian University of Life Sciences, Kreutzwaldi 1, 51006 Tartu, Estonia; 6 Indian Head Research Farm, Agriculture and Agri-Food Canada, Indian Head, SK, S0G 2K0, Canada; 7 Institute of Botany, University of Natural Resources and Life Sciences, Gregor-Mendel-Strasse 33, 1180 Vienna, Austria; 8 University of Basilicata, Via Ateneo Lucano 10, 85100 Potenza, Italy; 9 Direction de la Recherche Forestière, 2700 rue Einstein, Québec, QC, G1P 3W8, Canada; 10 Institute of Agro-Environmental & Forest Biology (IBAF), National Research Council (CNR), Via Marconi 2, Porano (TR) 05010, Italy; 11 Monash University, Australia

**Keywords:** Abiotic stress, *A–C*_i_ curve, mesophyll conductance, meta-analysis, photosynthesis, poplar

## Abstract

Mesophyll conductance (*g*_m_) determines the diffusion of CO_2_ from the substomatal cavities to the site of carboxylation in the chloroplasts and represents a critical component of the diffusive limitation of photosynthesis. In this study, we evaluated the average effect sizes of different environmental constraints on *g*_m_ in *Populus* spp., a forest tree model. We collected raw data of 815 *A–C*_i_ response curves from 26 datasets to estimate *g*_m_, using a single curve-fitting method to alleviate method-related bias. We performed a meta-analysis to assess the effects of different abiotic stresses on *g*_m_. We found a significant increase in *g*_m_ from the bottom to the top of the canopy that was concomitant with the increase of maximum rate of carboxylation and light-saturated photosynthetic rate (*A*_max_). *g*_m_ was positively associated with increases in soil moisture and nutrient availability, but was insensitive to increasing soil copper concentration and did not vary with atmospheric CO_2_ concentration. Our results showed that *g*_m_ was strongly related to *A*_max_ and to a lesser extent to stomatal conductance (*g*_s_). Moreover, a negative exponential relationship was obtained between *g*_m_ and specific leaf area, which may be used to scale-up *g*_m_ within the canopy.

## Teaser

Meta-analysis of mesophyll conductance in response to environmental constraints in *Populus* spp. showed significant effects of light gradient, soil moisture, and nitrogen but no response to copper and growth [CO_2_].

## Introduction

Carbon assimilation in plants is importantly determined by the diffusion efficiency of CO_2_ from the atmosphere to the site of carboxylation. The rate of CO_2_ diffusion is affected by two main diffusion limitations. The first limitation controls the CO_2_ flux from the atmosphere to the sub-stomatal cavities through the stomata and is characterized by stomatal conductance (*g*_s_). The second limitation determines the diffusion of CO_2_ from the substomatal cavities to the sites of carboxylation in the chloroplasts and is characterized by mesophyll conductance (*g*_m_). *g*_m_ is composed of gaseous and liquid phase resistances (Flexas *et al.*, 2008; Evans *et al.*, 2009; Niinemets *et al.*, 2009). CO_2_ diffusion inside the leaves is complex, facing a series of structural barriers coupled with biochemical regulation. It has been shown that *g*_m_ is typically limited by liquid phase conductance both in species with soft mesophytic leaves and in species with tough xerophytic leaves (Tosens *et al.*, 2012*a*, *b*; Tomás *et al.*, 2013). The liquid phase is a multicomponent pathway that involves the mesophyll cell wall thickness and porosity, the plasmalemma, the chloroplast envelope, the chloroplast thickness, and the mesophyll surface area exposed to intercellular air spaces per unit of leaf area (Evans *et al.*, 2009; Tosens *et al.*, 2012*b*; Tomás *et al.*, 2013). After extensive study during the past two decades, *g*_m_ is now widely accepted as a critical limiting factor to photosynthesis, which has to be considered in characterizing plant carbon gain potentials and responses to future climate change (Evans *et al.*, 2009; Niinemets *et al.*, 2009, 2011; Flexas *et al.*, 2016).

Mesophyll conductance has been shown to respond to environmental stress and may govern functional plasticity of photosynthesis and plant fitness under limited resources (Galle *et al.*, 2009; Barbour *et al.*, 2010; Buckley and Warren, 2014; Théroux Rancourt *et al.*, 2015; Flexas *et al.*, 2016; Shrestha *et al.*, 2018). However, recent findings on the response of *g*_m_ to abiotic stress are conflicting and inconclusive, demonstrating the complex nature of *g*_m_ variation (Flexas *et al.*, 2008; Niinemets *et al.*, 2009; Zhou *et al.*, 2014; Shrestha *et al.*, 2018). This suggests that the environmental and species-specific responses (and consequently the level of acclimation) of *g*_m_ to growth conditions should be considered in predicting plant performance in the field. Among the contrasting environmental responses, growth temperature may (Warren, 2008; Silim *et al.*, 2010) or may not (Dillaway and Kruger, 2010; Benomar *et al.*, 2018) affect *g*_m_. Similarly, the increase in soil nitrogen may (Warren, 2004; Shrestha *et al.*, 2018; Xu *et al.*, 2020; Zhu *et al.*, 2020) or may not (Bown *et al.*, 2009) stimulate *g*_m_. The magnitude of decrease in *g*_m_ under water stress and low light differed among studies (Warren *et al.*, 2003; Niinemets *et al.*, 2006; Montpied *et al.*, 2009; Bögelein *et al.*, 2012; Tosens *et al.*, 2012*a*; Zhou *et al.*, 2014; Peguero-Pina *et al.*, 2015; Théroux Rancourt *et al.*, 2015). These discrepancies among studies result in part from (i) the absolute changes in structural and biochemical traits controlling *g*_m_, as well as from changes in the relative contribution of these traits (Marchi *et al.*, 2008; Tomás *et al.*, 2013), and (ii) the level of coordination between *g*_m_, *g*_s_, and leaf specific hydraulic conductivity (*K*_L_) (Flexas *et al.*, 2013; Théroux-Rancourt *et al.*, 2014; Xiong *et al.*, 2017). Given the complex interplay between different factors controlling *g*_m_, it is important to examine its acclimation at the genus and species level to gain a general insight into the mechanistic basis of changes in *g*_m_.

Five methods exist to estimate *g*_m_: (i) chlorophyll fluorescence coupled to gas exchange (Harley *et al.*, 1992), (ii) carbon isotope discrimination coupled to gas exchange (initially developed by Evans *et al.*, 1986), (iii) oxygen isotope discrimination (Barbour *et al.*, 2016), (iv) *A*–*C*_i_ curve fitting (Ethier and Livingston, 2004; Sharkey *et al.*, 2007), and (v) 1D modeling of *g*_m_ from leaf structural characteristics (Evans *et al.*, 2009; Tosens *et al.*, 2012*b*; Tomás *et al.*, 2013). All of these methods are based on specific assumptions and each one has its limitations (Flexas *et al.*, 2013; Tosens and Laanisto, 2018). The standard deviation of the estimate of *g*_m_ may vary from 10% to 40%, which may limit our understanding of *g*_m_ acclimation to growth conditions, particularly when the variation between treatments or studies is less than the error of estimates (Sun *et al.*, 2014a).

*Populus* spp., model crops in forestry characterized by high yield potential, have been the subject of numerous studies to understand the physiological response to environmental factors but research is still necessary to make assessment of effects sizes and to make generalizations (Larocque *et al.*, 2013). A general understanding of the CO_2_ pathway through mesophyll and how it is affected by environmental factors would be beneficial in the effort to (i) accurately predict canopy photosynthesis under different environmental conditions, particularly under warmer and drier climate, and improve global carbon assimilation models, and (ii) effectively select more resilient and productive cultivars for wood and bioenergy. In poplar plantations, organic amendments like biosolids and pig slurry are used to increase growth rate at a low cost (Paniagua *et al.*, 2016). These amendments are rich in copper, the effect of which on photosynthetic activity, growth, and nutrient uptake has been well-documented in *Populus* spp. (Tognetti *et al.*, 2004; Borghi *et al.*, 2008; Pietrini *et al.*, 2017). In addition, poplar is a good candidate for environmental use in phytofiltration of contaminated water in agriculture lands, where copper is a major contaminant due to the large use of copper sulfate as a fungicide and in weed control (Fischerová *et al.*, 2006; Marmiroli *et al.*, 2011).

Substantial data of *A–C*_i_ response curves in the literature have been used to estimate photosynthetic parameters, not to estimate *g*_m_, and such compiled dataset would provide a basis to make such assessments of the response of *g*_m_ to the environment. In this study, we compiled 815 *A*–*C*_i_ response curves from 26 datasets of different poplar species and hybrids (Table 1). Published *A–C*_i_ curve-fitting approaches differ broadly regarding the rectangularity of the hyperbola, segmentations of the model of photosynthesis, and determination of the transition value of CO_2_ from carboxylation to electron transport (Harley *et al.*, 1992; Ethier and Livingston, 2004; Manter and Kerrigan, 2004; Dubois *et al.*, 2007; Sharkey *et al.*, 2007; Pons *et al.*, 2009; Gu *et al.*, 2010). These approaches led to different fitted values (Miao *et al.*, 2009; Sun *et al.*, 2014a). Although *A–C*_i_ curve fitting is unreliable for species with large *g*_m_, it can provide results similar to those obtained from direct measurements for species with medium to low *g*_m_ (Niinemets *et al.*, 2005, 2006; Warren, 2006; Qiu *et al.*, 2017; Xu *et al.*, 2020). Using the compiled *A–C*_i_ response curves, we performed curve fitting using a single method (Ethier and Livingston, 2004) to alleviate the fitting method bias and to obtain uniformed estimates of *g*_m_, maximum rate of carboxylation (*V*_cmax_) and rate of electron transport (*J*). We further collected related variables like leaf nitrogen content, stomatal conductance, and specific leaf area (SLA) when data were available. Our main goal was to find trends in the response of mesophyll conductance to prevalent abiotic stressors and to examine the relationship between *g*_m_ and other leaf traits. We believe that a meta-analytical approach to analyse the accumulated data on the diffusion of CO_2_ through the mesophyll diffusion pathway in relation to other photosynthesis-related traits provides key insights into the different controls on mesophyll conductance and into the environmental plasticity of mesophyll conductance. We aim to contribute to the efforts of improving poplar photosynthetic efficiency in poplar breeding programs, and to improve modelling of global carbon assimilation of biomass and bioenergy crops under climate change.

**Table 1.** List of dataset sources used in the meta-analysis

Author	*Populus* species or hybrid parents	Number of genotypes	Treatment	Provenance of plant material	Growth Environment	Number of curves
Attia *et al.* (2015)	*P. balsamifera* L.	3	N/A	Canada	Growth chamber	15
	*P. simonii* Carrière					
	*P. balsamifera* L. *× P. simonii* Carrière					
Benomar *et al.* (https://doi.org/10.5061/dryad.9cnp5hqhp)	*P. maximowiczii* A. Henry *× P. balsamifera* L.	2	Water stress	Canada	Growth chamber	12
Benomar (2012)	*P. maximowiczii* A. Henry *× P. balsamifera* L.	2	Spacing and canopy level	Canada	Plantation	52
	*P. balsamifera* L. *× P. trichocarpa* Torr. & A. Gray					
Benomar *et al.* (2019)	*P. maximowiczii* A. Henry *× P. balsamifera* L.	2	Temperature and nitrogen	Canada	Growth chamber	23
	*P. maximowiczii* A. Henry *× P. nigra* L.					
Borghi *et al.* (2007)	*P. × euramericana* (*P. deltoides* W. Bartram *× P. nigra* L.) (clone Adda)	1	Copper	Italy	Growth chamber	21
Borghi *et al.* (2008)	*P. alba* L.	2	Copper	Italy	Growth chamber	18
	*P. × Canadensis* (*P. nigra* L. *× P. deltoides* W. Bartram)					
Calfapietra *et al.* (2005)	*P. × euramericana* (*P. deltoides* W. Bartram *× P. nigra* L.)	1	Nitrogen and atmospheric CO_2_ and canopy level	Italy	Plantation	60
Castagna *et al.* (2015)	*P. × canadensis* (*P. nigra* L. *× P. deltoides* W. Bartram)	2	Ozone and cadmium soil contamination	Italy	Greenhouse	16
	*P. deltoides* W. Bartram *× P. maximowiczii* A. Henry					
Di Baccio *et al.* (2009)	*P. × euramericana* (*P. deltoides* W. Bartram × *P. nigra* L.) (clone i-214)	1	Zinc soil contamination	Italy	Growth chamber	12
Elferjani *et al.* (2016)	*P. trichocarpa* Torr. & A. Gray *× P. balsamifera* L. (clone 747215)	4	Latitudinal gradient	Canada	Plantation	24
	*P. balsamifera* L. *× P. maximowiczii* A. Henry (clones 915004 and 915005)					
	*P. maximowiczii* A. Henry *× P. balsamifera* L. (clone 915319)					
Li *et al.* (2013)	*P. euphratica* Oliv.	1	Ground water availability	China	In field under shelter (lysimeter)	9
Merilo *et al.* (2010)	*P. nigra* L.	2	Atmospheric CO_2_ (FACE) and nitrogen and canopy level	Italy	Plantation	104
	*P. alba* L.					
Niinemets *et al.* (1998)	*P. tremula* L.	1	Canopy level	Estonia	Natural forest stands	14
Ripullone *et al.* (2003)	*P. × euramericana* (*P. deltoides* W. Bartram *× P. nigra* L.) (clone i-214)	1	Nitrogen	Italy	Greenhouse	14
Ryan *et al.* (2009)	*P. deltoides* W. Bartram *× P. trichocarpa* Torr. & A. Gray	2	Ozone	United Kingdom	Greenhouse	118
Silim *et al.* (2010)	*P. balsamifera* L.	1	Habitat and growth temperature	Canada	Greenhouse	30
Soolanayakanahally *et al.* (2009)	*P. balsamifera* L.	1	Latitudinal gradient	Canada	Greenhouse	72
Théroux-Rancourt *et al.* (https://doi.org/10.5061/dryad.7sqv9s4s0)	*P. deltoides* W. Bartram *× P. nigra* L. (clone 3570)	8	Water stress	Canada	Greenhouse	38
	*P. maximowiczii* A. Henry *×* (*P. deltoides* W. Bartram *× P. trichocarpa* Torr. & A. Gray) (clones 505372 and 505508)					
	*P. maximowiczii* A. Henry *× P. trichocarpa* Torr. & A. Gray (clone 750361)					
	*P. maximowiczii* A. Henry *× P. balsamifera* L. (clones 915302, 915313, 915318)					
	(*P. deltoides* W. Bartram *× P.* nigra L.) *× P. trichocarpa* Torr. & A. Gray (clone 915508)					
Théroux-Rancourt *et al.* (2014)	*Assiniboine:* [(*P. × ‘Walker’: P. deltoides* W. Bartram *× P. × petrowskiana* R. I. Schrod. ex Regel) × male parent unknown]	2	N/A	Canada	Greenhouse and growth chamber	**3**
	Okanese [(*P.×*’Walker’) *× P.× petrowskiana* R. I. Schrod. ex Regel]					
Théroux-Rancourt *et al*. (2015)	(*P. maximowiczii* A. Henry)*×* (*P. deltoidesW. Bartram × P. trichocarpa* Torr. & A. Gray)	5	N/A	Canada	Greenhouse and growth chamber	12
	*P. maximowiczii* A. Henry *× P. balsamifera* L.					
	‘Walker’ [*P. deltoides* W. Bartram *×* (*P. laurifolia Ledeb. × P. nigra L.*)] *× P. deltoides* W. Bartram					
	‘Walker’ *× P. petrowskyana* Schr.					
	*P. balsamifera* L.					
Tissue and Lewis (2010)	*P. deltoides* W. Bartram	1	Phosphorous and atmospheric CO_2_	Australia	Growth chamber	76
Tognetti *et al.* (https://doi.org/10.5061/dryad.w3r2280qq)	*P. × euramericana* (*P. nigra* L. *× P. deltoides* W. Bartram) (clone i-214)		Zinc soil contamination	Italy	Greenhouse	24
Tognetti *et al.* (2004)	*P. deltoides* W. Bartram *× P. maximowiczii* A. Henry	2	Heavy metals	Italy	Greenhouse	24
	*P. × euramericana* (*P. deltoides* W. Bartram *× P. nigra* L.) (clone i-214)					
Tosens *et al.* (2012a)	*P. tremula* L.	1	Light and water stress	Estonia	Growth chamber	8
Velikova *et al.* (2011)	*P. nigra* L.	20	Nickel soil contamination	Italy	Growth chamber (climate chamber)	16
Xu *et al.* (2020)	*P. × euramericana* (*P. deltoides* W. Bartram *× P. nigra* L.) (cv. ‘74/76’)	1	Nitrogen and ozone	China	Growth chamber	6

## Materials and methods

### Data collection

Data were collected by a web search in Web of Science, Scopus, and Google Scholar using the following key words: (‘*Populus*’ or ‘poplar’ or ‘hybrid poplar’ or ‘aspen’) and (‘*V*_cmax_’ or ‘maximum rate of electron transport (*J*_max_)’ or ‘mesophyll conductance’). At this step, the abstract of every item was checked to confirm the paper is actually about *g*_m_. Then, we looked at the ‘Materials and methods’ section of selected papers where *A*–*C*_i_ response curves of *Populus* spp. were measured.

To get raw data of *A*–*C*_i_ response curves, we contacted the corresponding authors or co-authors of the targeted studies by e-mail and via ResearchGate. We obtained 23 datasets from published studies and three datasets from Benhomar, Tognetti and Théroux-Rancourt studies (Table 1; datasets available at Dryad Digital Repository). Collectively, they provided a total of 815 *A*–*C*_i_ response curves.

The total data of 72 genotypes were collected from measurements on plants growing in plantations (five studies), or under controlled conditions (greenhouse or growth chamber set-ups; 21 studies) with optimal and stressful conditions (Table 1). After compiling all *A*–*C*_i_ curves, the quality of the data was assessed based on the following criteria: (i) only curves with at least two points in the saturation region (*J* region) were retained; (ii) only fitted curves with *P-*value <0.05 using the method of Ethier and Livingston (2004) were retained, and consequently 65 curves that did not meet these conditions were removed; and (iii) based on the literature, *g*_m_ values in *Populus* spp. using at least two methods simultaneously never exceeded 1 mol m^−2^ s^−1^ (Singsaas *et al.*, 2004; Flexas *et al.*, 2008; Velikova *et al.*, 2011; Tosens *et al.*, 2012*a*; Théroux-Rancourt *et al.*, 2014; Momayyezi and Guy, 2017; Xu *et al.*, 2020). Then, *g*_m_ values >1 mol m^−2^ s^−1^ were considered as non-available data (94 entries), and *V*_cmax_ and *J* values were retained for further analyses.

### Data subsets

To examine the effect of a given abiotic factor on *g*_m_, we estimated that a minimum of three studies is necessary to have reliable conclusions, regardless of the genotype used, except copper for which only two studies were examined because they had been conducted under the same experimental conditions. Then, we could come up with subsets of data that focused on the same variable and performed analyses on them separately (identified in the column ‘Treatment’ in Table 1). Our first goal was to examine the effect of variations in these factors on *g*_m_, light-saturated photosynthetic rate (*A*_max_), *g*_s_, *J*, *V*_cmax_, and in a second step, the relationships between *g*_m_ and other photosynthetic characteristics (*A*_max_, *g*_s_, *J*, *V*_cmax_). The data subsets included the following environmental factors:

• Canopy level: four studies addressed the photosynthetic activity of leaves at the bottom, middle and top of trees (Niinemets *et al.*, 1998; Calfapietra *et al.*, 2005; Merilo *et al.*, 2010; Benomar, 2012).
• Atmospheric CO_2_: we examined the response of trees to elevated atmospheric CO_2_ from the studies of Calfapietra *et al.* (2005), Merilo *et al.* (2010) and Tissue and Lewis (2010). We considered 370 ppm as the control treatment in the three studies, while the elevated CO_2_ was 550 ppm of CO_2_ for the studies of Calfapietra *et al.* (2005) and Merilo *et al.* (2010), and 700 ppm for the study of Tissue and Lewis (2010).
• Copper (Cu) stress: datasets from the studies of Borghi *et al.* (2007) and Borghi *et al.* (2008) were used to examine the response of poplar trees to contamination of the substrate with Cu. Treatments were assigned to three levels of Cu: 0 (0–0.4 µM), 20 (20–25 µM) and 75 (75–100 µM).
• Soil nitrogen (N) content: high *vs*. low soil N content treatments were reported in four studies: Ripullone *et al.* (2003), Calfapietra *et al.* (2005), Benomar *et al.* (2018) and Xu *et al.* (2020). In the study of Merilo *et al.* (2010), the authors showed that no effect of nitrogen fertilization was observed due to high background nutrient availability in the plantation site.
• Soil moisture: water status of trees was assessed and data from four studies were classified into two treatments: control (optimal watering) *vs*. water deficit (Li *et al.*, 2013; Tosens *et al.*, 2012*a*; Théroux-Rancourt (data available at Dryad Digital Repository: https://doi.org/10.5061/dryad.7sqv9s4s0); Benomar (data available at Dryad Digital Repository: https://doi.org/10.5061/dryad.9cnp5hqhp).

For Xu *et al.* (2020), we extracted data from the article (means and standard errors) and generated three replicates assuming a normal distribution using the SURVEYSELECT procedure of SAS (version 9.4; SAS Institute, Cary, NC, USA). The reason is that the authors used the same curve fitting approach (Ethier and Livingston, 2004) the we used in this meta-analysis study (Table 1).

For studies with two or more investigated factors, we considered the different levels of the factor of interest and the control level of the rest of the factors to avoid between-factor interaction effects on the results. For example, in Calfapietra *et al.* (2005), trees were subject to different levels of N and CO_2_; when we focused on the effect of N, we selected trees exposed to ambient CO_2_ only (control).

### Curve analysis

Mesophyll conductance and photosynthetic capacity variables, *V*_cmax_ and *J*, were estimated by fitting *A*–*C*_i_ curve with the non-rectangular hyperbola version (Ethier and Livingston, 2004) of the biochemical model of C_3_ plants (Farquhar *et al.*, 1980). This method was calibrated for low *g*_m_ species (<0.3 mol m^−2^ s^−1^) and its accuracy is similar to estimates using the chlorophyll fluorescence method and online carbon ^13^C isotope discrimination (Niinemets *et al.*, 2005, 2006; Ethier *et al.*, 2006; Tomás *et al.*, 2013; Qiu *et al.*, 2017; Xu *et al.*, 2020). The model was fitted using non-linear regression techniques (Proc NLIN, SAS) following Dubois *et al.* (2007) and Sun *et al.* (2014a).

Briefly, the net assimilation rate (*A*_n_) is given as:


$$\begin{array}{l} A_{n}=min\;\left\{A_{c},\;A_{j}\right\} \end{array}$$ (1)

with


$$\begin{array}{l} A_{c}=V_{cmax}\frac{\left(C_{c}-\Gamma^{*}\right)}{C_{c}+K_{c}\left(1+\frac{O}{K_{o}}\right)}-R_{day} \end{array}$$ (2)

$$\begin{array}{l} A_{j}=J\;\frac{C_{c}-\Gamma^{*}}{4\left(C_{c}+2\Gamma^{*}\right)}-R_{day} \end{array}$$ (3)

$$\begin{array}{l} C_{c}=C_{i}-\frac{A_{n}}{g_{m}} \end{array}$$ (4)

where *A*_c_ is the Rubisco-limited rate of CO_2_ assimilation (µmol m^−2^ s^−1^), *A*_j_ is the RuBP-limited rate of CO_2_ assimilation (µmol m^−2^ s^−1^), *V*_cmax_ is the maximum rate of carboxylation (µmol m^−2^ s^−1^), *O* is the partial atmospheric pressure of O_2_ (mmol mol^−1^)_,_Γ^*^ is the CO_2_ compensation point in the absence of mitochondrial respiration, *R*_day_ is mitochondrial respiration in the light (µmol CO_2_ m^−2^ s^−1^), *C*_c_ is the chloroplast CO_2_ (µmol mol^−1^), *C*_i_ is the intercellular air space concentration of CO_2_ (µmol mol^−1^), *K*_c_ (µmol mol^−1^) and *K*_o_ (mmol mol^−1^) are the Michaelis–Menten constants of Rubisco for CO_2_ and O_2_, respectively, and *J* is the rate of electron transport (µmol m^−2^ s^−1^). The values at 25 °C used for *K*_c_, *K*_o_, and Γ^*^ were 272 µmol mol^−1^, 166 mmol mol^−1^ and 37.4 µmol mol^−1^, respectively (Sharkey *et al.*, 2007), and their temperature dependencies were as in Sharkey *et al.* (2007).

In four datasets, measurements were carried out under a temperature that was different from the reference (25 °C). In this case, *V*_cmax_ and *J* were normalized to 25 °C using the model of Kattge and Knorr (2007), which integrates the acclimation to growth temperature. However, the actual values of *V*_cmax_ and *J* were more often significant compared with normalized values, and this was true using both ANOVA and regression analyses.

### Quantitative limitations analysis

The stomatal conductance (*L*_s_), mesophyll conductance (*L*_m_), and biochemical (*L*_b_) relative limitations to photosynthesis were estimated following Grassi and Magnani (2005) as modified by Tomás *et al.* (2013):


$$\begin{array}{l} L_{s}=\frac{\left({}^{g_{tot}}╱{}_{g_{sc}}\;\right){}^{\partial A_{c}}╱{}_{\partial C_{c}}\;}{g_{tot}+{}^{\partial A_{c}}╱{}_{\partial C_{c}}\;} \end{array}$$ (5)

$$\begin{array}{l} L_{m}=\frac{\left({}^{g_{tot}}╱{}_{g_{m}}\;\right){}^{\partial A_{c}}╱{}_{\partial C_{c}}\;}{g_{tot}+{}^{\partial A_{c}}╱{}_{\partial C_{c}}\;} \end{array}$$ (6)

$$\begin{array}{l} L_{b}=\frac{g_{tot}}{g_{tot}+{}^{\partial A_{c}}╱{}_{\partial C_{c}}\;} \end{array}$$ (7)

where *g*_tot_ is the total CO_2_ conductance and *g*_sc_ is the stomatal conductance to CO_2_ (*g*_sc_=*g*_sw_/1.6).


$$\begin{array}{l} g_{tot}=\frac{1}{\frac{1}{g_{sc}}+\frac{1}{g_{m}}} \end{array}$$ (8)

$$\begin{array}{l} \frac{\partial A_{c}}{\partial C_{c}}=\frac{V_{cmax}\left(\Gamma^{*}+k_{c}\left(1+{}^{O}╱{}_{k_{o}}\;\right)\right)}{{\left(C_{c}+k_{c}\left(1+{}^{O}╱{}_{k_{o}}\;\right)\right)}^{2}} \end{array}$$ (9)

where $\frac{\partial A_{c}}{\partial C_{c}}$ is the first derivative of *A*_c_ with respect to *C*_c_.

Factors for which *A*_max_ changed significantly (canopy level, soil nitrogen, and soil moisture), the absolute contribution of stomatal conductance limitation (*S*_L_), mesophyll conductance (MC_L_), and biochemical photosynthetic capacity limitation (*B*_L_) to observed change of *A*_max_ were estimated following Grassi and Magnani (2005):


$$\begin{array}{l} \frac{dA_{max}}{A_{max}}=S_{L}+MC_{L}+B_{L}=L_{s}\frac{dg_{sc}}{g_{sc}}+L_{m}\frac{dg_{m}}{g_{m}}+L_{b}\frac{dV_{cmax}}{V_{cmax}} \end{array}$$ (10)

where $\frac{dA_{max}}{A_{max}}$ is the difference of *A*_max_ between the reference and the other treatments (within each factor) divided by *A*_max_ of the reference.

### Statistical analyses

Data analysis assessing the effect of the environmental factors on *g*_m_ and the relationship between *g*_m_ and the other traits were carried out using SAS version 9.4.

For studies that focused on nitrogen, soil moisture, CO_2_, canopy level and copper, the effect of treatments on light-saturated photosynthetic rate (*A*_max_), *g*_m_, and *g*_sw_ was assessed separately for each response variable, using mixed model analyses of variance of the primary data (Riley *et al.*, 2010; Mengersen *et al.*, 2013). ‘Treatment’ was the fixed effect while ‘study’ and ‘genotype’ nested within study were the random effects. The number of replicates was not necessarily balanced across treatments. The assumptions of normality of the residuals and homogeneity of variance were verified, and a log-transformation was made when necessary.

## Results

**Fig. 1.**
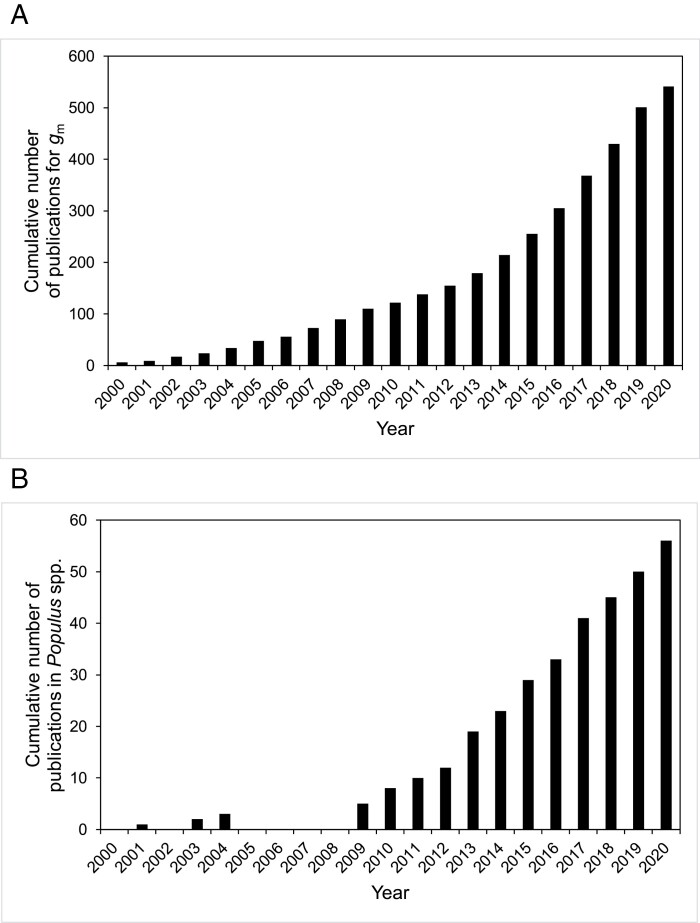
Cumulative number of published studies for mesophyll conductance (*g*_m_) between the years 2000 and 2020 (A), and cumulative number of published studies for mesophyll conductance (*g*_m_) in *Populus* spp. between the years 2001 and 2020 (B). The number of publications was determined using keywords (e.g. *g*_m_ and *Populus*) through database search available at the Web of Science Core Collection (https://clarivate.com/webofsciencegroup/solutions/web-of-science-core-collection/).

The number of studies on mesophyll conductance has rapidly increased since 2000, and more remarkably since 2013 (Fig. 1A), suggesting a growing interest among plant ecophysiologists in understanding the role of *g*_m_ in photosynthesis. This pattern was very similar to the increase of publication number on mesophyll conductance in *Populus* spp. (Fig. 1B).

### Canopy level

**Fig. 2.**
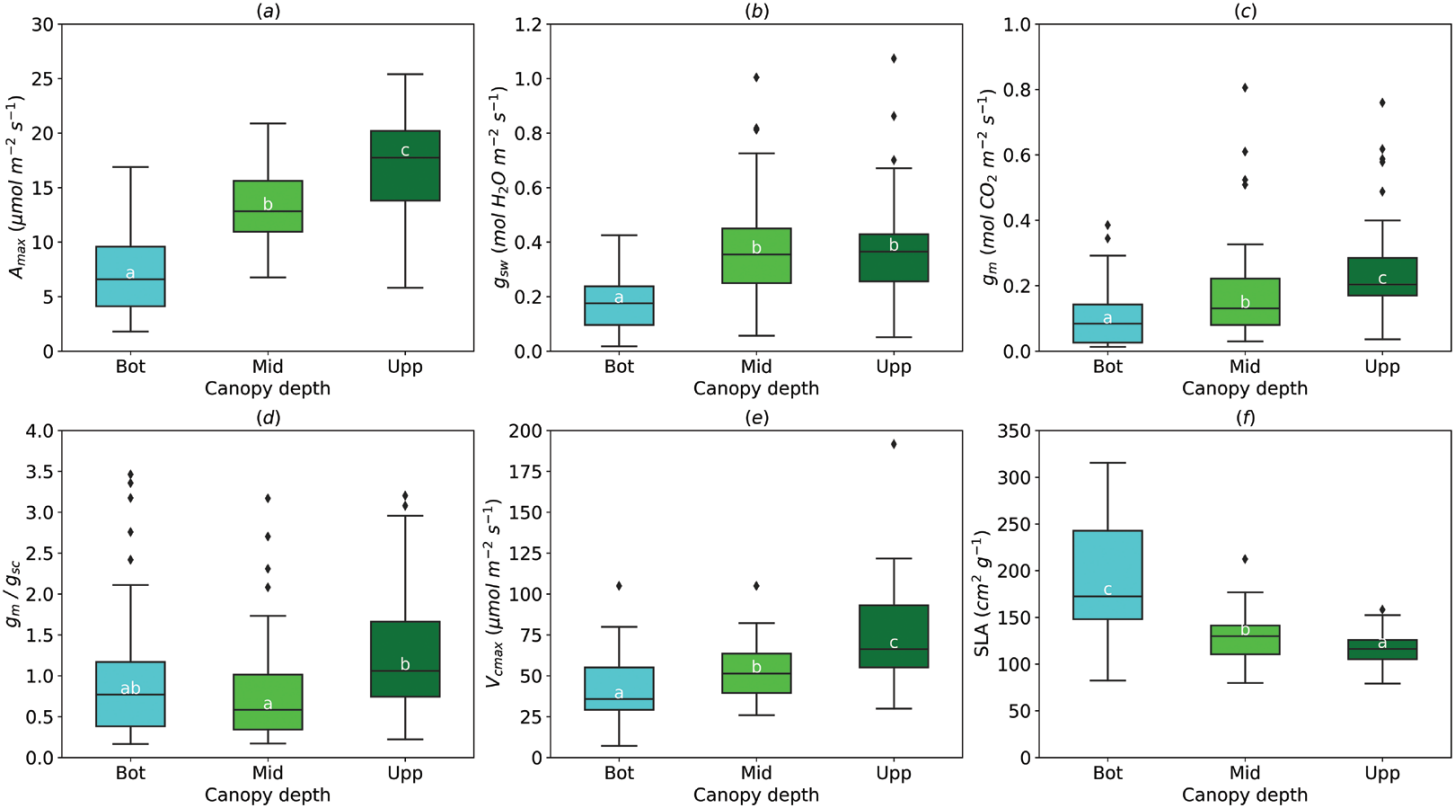
Effect of the leaf position in the canopy (Bot, bottom; Mid, middle; Upp, upper) on light-saturated photosynthetic rate (*A*_max_, A), stomatal conductance (*g*_sw_, B), mesophyll conductance (*g*_m_, C), *g*_m_/*g*_sc_ ratio (D), maximum rate of carboxylation (*V*_cmax_, E), and specific leaf area (*SLA*, F). For *g*_m_/*g*_sc_ ratio, *g*_sw_ for water (mol H_2_O m^−2^ s^−1^) was divided by 1.6 to obtain *g*_sc_ (mol CO_2_ m^−2^ s^−1^). The horizontal line inside the boxes marks the median for the observations, the box ends indicate the upper (third) to lower (first) quartiles of the value ranges, and the whiskers indicate the highest and lowest observations. Means having the same letters are not significantly different at α=0.05 (number of studies=4, number of genotypes=6).

Light-saturated photosynthetic rate at an ambient CO_2_ concentration (380–400 µmol mol^−1^), *A*_max_, significantly increased from 7.19±0.44 µmol m^−2^ s^−1^ on average at the bottom leaves to 13.15±0.45 µmol m^−2^ s^−1^ at the mid-canopy, to 16.29±0.53 µmol m^−2^ s^−1^ at the upper canopy (Fig. 2A; Supplementary Table S1). Similar to *A*_max_, *g*_m_ had an ascending pattern, from the bottom (0.12±0.01 mol CO_2_ m^−2^ s^−1^) to the top of the canopy (0.24±0.02 mol m^−2^ s^−1^) (Fig. 2C). Stomatal conductance (*g*_sw_) was the lowest at the bottom canopy (0.17±0.01 mol H_2_O m^−2^ s^−1^) and then increased to 0.36±0.02 mol H_2_O m^−2^ s^−1^ at the mid and upper canopy (Fig. 2B). The *g*_m_/*g*_sc_ ratio was significantly greater at the upper canopy (1.17±0.11), compared with the mid-canopy leaves (0.88±0.09) and was not different everywhere else (Fig. 2D). *V*_cmax_ increased similarly to *A*_max_ and *g*_m_ from the bottom to the top of the canopy (Fig. 2E); however, SLA had an opposite trend (Fig. 2F).

### Atmospheric CO_2_

**Fig. 3.**
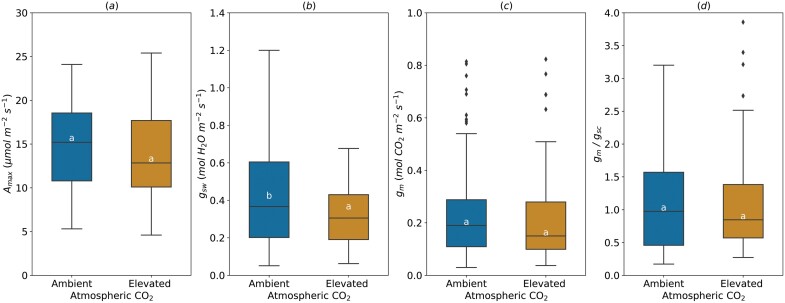
Effect of the atmospheric CO_2_ concentration on light-saturated photosynthetic rate (*A*_max_, A), stomatal conductance (*g*_sw_, B), mesophyll conductance (*g*_m_, C), and *g*_m_/*g*_sc_ ratio (D). For *g*_m_/*g*_sc_ ratio, *g*_sw_ for water (mol H_2_O m^−2^ s^−1^) was divided by 1.6 to obtain *g*_sc_ (mol CO_2_ m^−2^ s^−1^). The horizontal line inside the boxes marks the median for the observations, the box ends indicate the upper (third) to lower (first) quartiles of the value ranges, and the whiskers indicate the highest and lowest observations. Means having the same letters are not significantly different at α=0.05 (number of studies=3, number of genotypes=4).

Increased atmospheric CO_2_ had no effect on average *A*_max_ (14.43±0.60 µmol m^−2^ s^−1^), *g*_m_ (0.21±0.02 mol m^−2^ s^−1^) and *g*_m_/*g*_sc_ (1.09±0.11) (Fig. 3A–C). However, average *g*_sw_ was higher (0.40±0.03 mol H_2_O m^−2^ s^−1^) under ‘Ambient’, compared with ‘Elevated’ CO_**2**_ (0.32±0.02 mol H_2_O m^−2^ s^−1^) (Fig. 3B).

### Copper stress

**Fig. 4.**
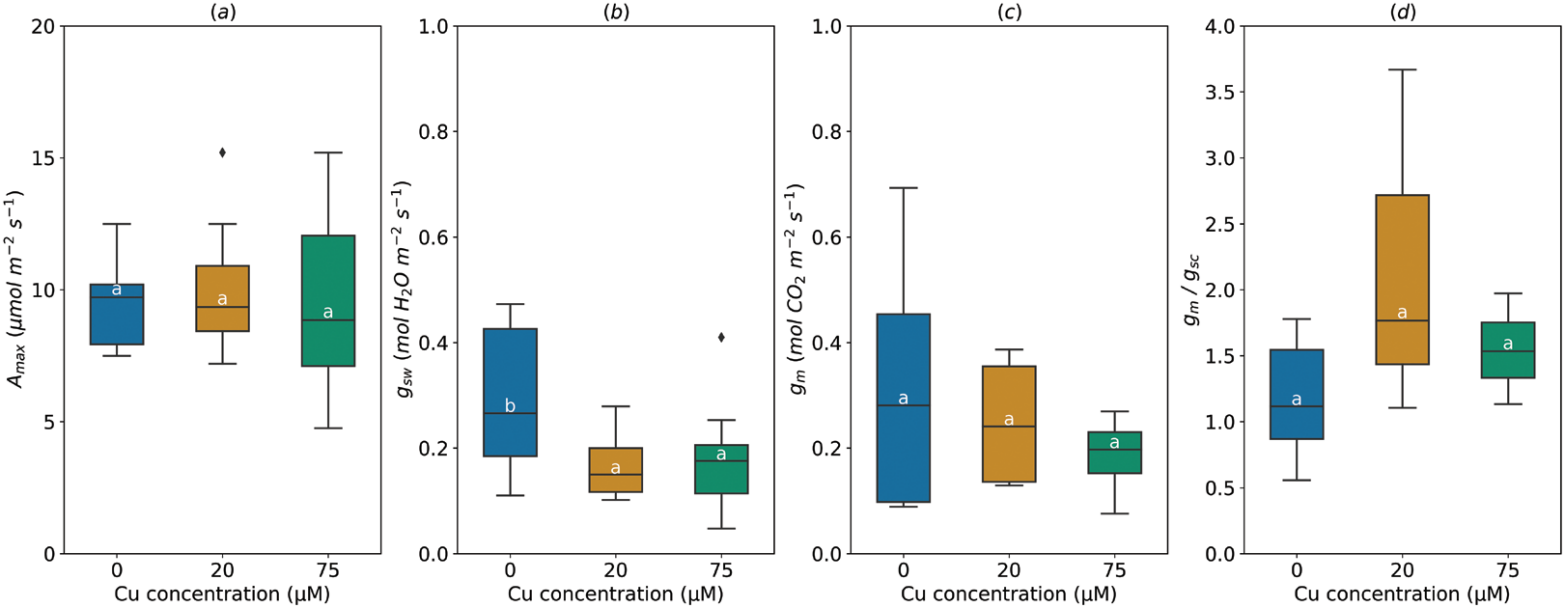
Effect of the soil copper (Cu) concentration on light-saturated photosynthetic rate (*A*_max_, A), stomatal conductance (*g*_sw_, B), mesophyll conductance (*g*_m_, C), and *g*_m_/*g*_sc_ ratio (D). For *g*_m_/*g*_s_ ratio, *g*_s_ for water (mol H_2_O m^−2^ s^−1^) was divided by 1.6 to obtain *g*_sc_ (mol CO_2_ m^−2^ s^−1^). The horizontal line inside the boxes marks the median for the observations, the box ends indicate the upper (third) to lower (first) quartiles of the value ranges, and the whiskers indicate the highest and lowest observations. Means having the same letters are not significantly different at α=0.05 (number of studies=2, number of genotypes=3).

*A*_max_ was not affected when soil Cu concentration increased from 0 to 20 or 75 µM (9.67±0.95 µmol m^−2^ s^−1^) (Fig. 4A). It should be noted that at the highest Cu level (75 µM), *A*_max_ ranged from 4 to 15 µmol m^−2^ s^−1^. Average *g*_sw_ significantly decreased under medium (20 µM, 0.17±0.02 mol H_2_O m^−2^ s^−1^) and high Cu treatment (75 µM, 0.18±0.03 mol m^−2^ s^−1^), compared with control treatment (Fig. 4B). Increasing Cu concentration in the soil did not affect *g*_m_ and the *g*_m_/*g*_sc_ ratio (Fig. 4C, D).

### Soil nitrogen

**Fig. 5.**
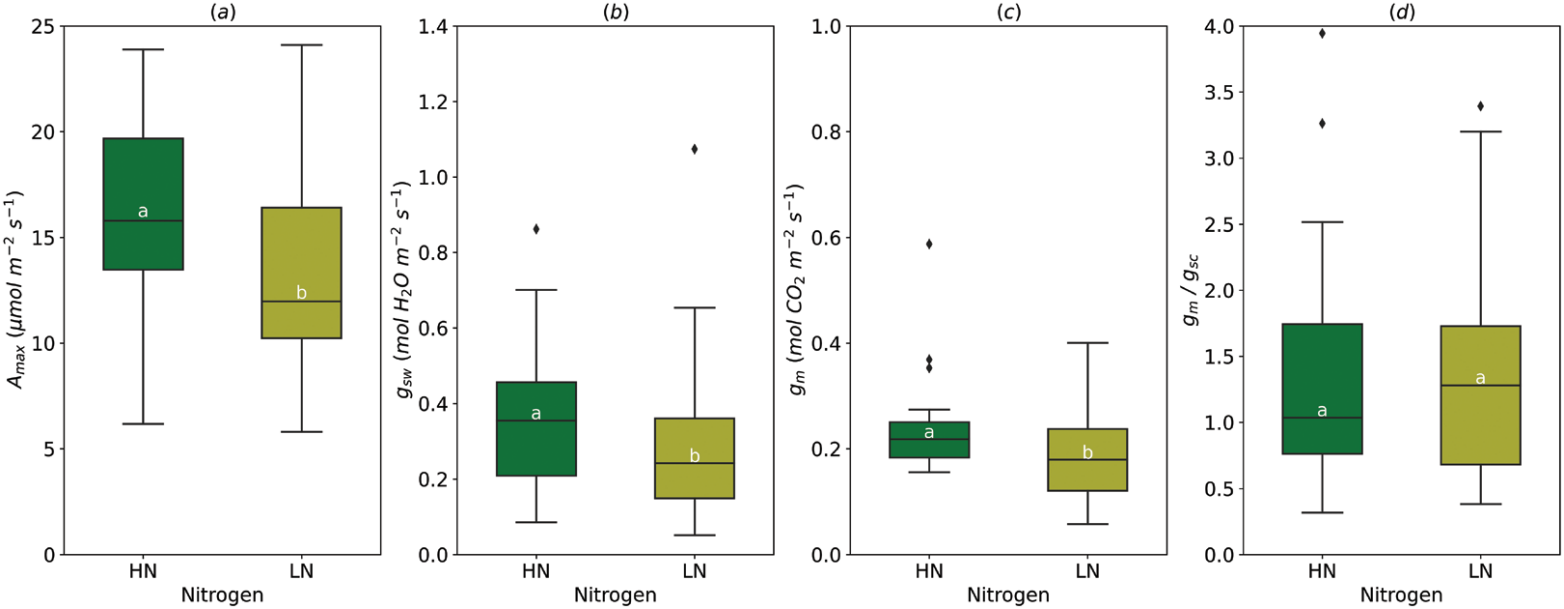
Effect of the soil nitrogen content (HN, high nitrogen; LN, low nitrogen) on light-saturated photosynthetic rate (*A*_max_, A), stomatal conductance (*g*_sw_, B), mesophyll conductance (*g*_m_, C), and *g*_m_/*g*_sc_ ratio (D). For *g*_m_/*g*_sc_ ratio, *g*_sw_ for water (mol H_2_O m^−2^ s^−1^) was divided by 1.6 to obtain *g*_sc_ (mol CO_2_ m^−2^ s^−1^). The horizontal line inside the boxes marks the median for the observations, the box ends indicate the upper (third) to lower (first) quartiles of the value ranges, and the whiskers indicate the highest and lowest observations. Means having the same letters are not significantly different at α=0.05 (number of studies=5, number of genotypes=7).

*A*_max_ was significantly greater (16.07±0.61 µmol m^−2^ s^−1^) under high soil nitrogen (HN, 250 kg N ha^−1^ y^−1^ in field study or 20 mM for pot study) compared with low nitrogen treatment (LN, 12.93±0.65 µmol m^−2^ s^−1^) (Fig. 5A). A high supply of nitrogen increased *g*_sw_ (from 0.29±0.03 in LN to 0.36±0.03 mol m^−2^ s^−1^ in HN) and *g*_m_ (from 0.19±0.02 to 0.23±0.02 to mol m^−2^ s^−1^), but had no effect on the *g*_m_/*g*_sc_ ratio (1.38±0.16 on average) (Fig. 5B–D).

### Soil moisture

**Fig. 6.**
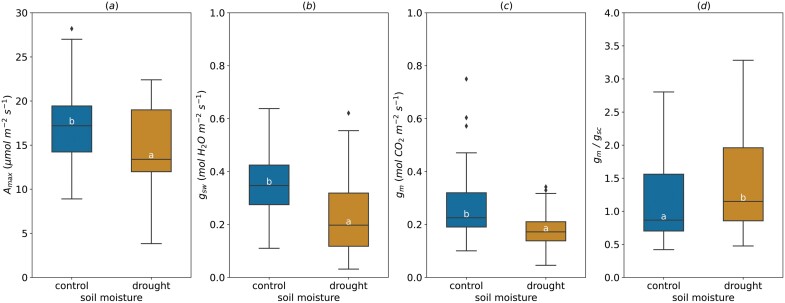
Effect of the soil moisture on light-saturated photosynthetic rate (*A*_max_, A), stomatal conductance (*g*_sw_, B), mesophyll conductance (*g*_m_, C), and *g*_m_/*g*_sc_ ratio (D). For *g*_m_/*g*_sc_ ratio, *g*_sw_ for water (mol H_2_O m^−2^ s^−1^) was divided by 1.6 to obtain *g*_sc_ (mol CO_2_ m^−2^ s^−1^). The horizontal line inside the boxes marks the median for the observations, the box ends indicate the upper (third) to lower (first) quartiles of the value ranges, and the whiskers indicate the highest and lowest observations. Means having the same letters are not significantly different at α=0.05 (number of studies=4, number of genotypes=13).

Average *A*_max_ decreased by drought (range of leaf predawn water potential under water deficit, Ψ _leaf_=−0.7 to −0.8, soil water content=10%), dropping from 17.13±0.71 µmol m^−2^ s^−1^ to 14.62± 0.91 µmol m^−2^ s^−1^ on average with the minimum value (3.83 µmol m^−2^ s^−1^) much lower than in watered trees (8.90 µmol m^−2^ s^−1^) (Fig. 6A). As expected, soil moisture deficit markedly altered *g*_sw_, decreasing its average value from 0.33±0.02 mol m^−2^ s^−1^ in control trees to 0.20±0.03 mol m^−2^ s^−1^ under drought conditions (Fig. 6B). Drought had the same effect on *g*_m_, but to a lesser extent than *g*_sw_. *g*_m_ decreased from 0.27±0.02 mol m^−2^ s^−1^ to 0.19±0.02 mol m^−2^ s^−1^ under soil moisture deficit (Fig. 6C). In addition, the *g*_m_/*g*_sc_ ratio increased by 37% when plants were subject to drought (Fig. 6D).

### Quantitative limitations

In general, photosynthetic rate was mostly limited by CO_2_ diffusion (up to 75%): stomatal limitation (*L*_s_) and mesophyll limitation (*L*_m_) (Table 2). Biochemical limitation (*L*_b_) of photosynthesis rate was relatively low. Higher atmospheric CO_2_ decreased biochemical limitation (16.62% to 14.73%) and increased mesophyll limitation (from 41.80% to 44.28%) while stomatal limitation remained unchanged. Within the canopy, stomatal and biochemical limitations were the greatest in the upper (47.79%) and the middle (16.05%) layers of the canopy, respectively (Table 2). The mesophyll conductance limitation was higher at the middle (50.47%) and the bottom (50.85%) than at the upper part of the canopy (40.51%). The decrease of *A*_max_ (58.04 %) from the top, as a reference, to the bottom of the canopy (calculated with Equation 10) was mostly caused by mesophyll (absolute limitation=29.48%), followed by stomatal (19.16%) limitation and to a lesser extent by *V*_cmax_ (5.11%). At the middle of the canopy, the decrease of *A*_max_ (21.58%) was mostly due to *g*_m_ (13.15%) and to a lesser extent to *V*_cmax_ (4.74%) while the contribution of *g*_sc_ was marginal (2.76%). The copper stress resulted in an increase of the stomatal limitation and a decrease in mesophyll and biochemical limitations. Change in soil nitrogen did not affect the status of the limitations. The decrease of *A*_max_ (20.11 %) under low soil nitrogen was mostly caused by *g*_sc_ (9.38%) and *g*_m_ (7.58%) and to a lesser extent by *V*_cmax_ (1.42%). Water stress increased stomatal limitation and decreased biochemical limitation but had no effect on mesophyll limitation (Table 2). Therefore, the observed decrease in *A*_max_ (21.02%) under water deficit was mainly due to stomatal (15.22 %) and mesophyll limitation (7.64 %).

**Table 2.** The relative limitation (%) of stomatal conductance, mesophyll conductance, and biochemistry to photosynthesis (for each treatment, the sum of relative limitations is 100%)

Factor	Treatment	Stomatal limitation (*L*_s_)	Mesophyll limitation (*L*_m_)	Biochemical limitation (*L*_b_)
Canopy level	Bottom	37.71 (2.11)	50.86 (2.68)	11.43 (1.22)
	Middle	33.46 (1.99)	50.48 (2.4)	16.06 (1.34)
	Upper	45.97 (2.18)	40.52 (1.98)	13.69 (1.09)
Atmospheric CO_2_	Ambient	41.57 (2.3)	41.8 (2.27)	16.62 (1.43)
	Elevated	40.97 (1.87)	44.29 (2.04)	14.74 (1.21)
Copper stress	0 μM Cu	42.42 (6.78)	31.17 (7.38)	26.43 (7.1)
	20 μM Cu	57.88 (4.96)	25.16 (6.7)	16.96 (2.71)
	75 μM Cu	59.13 (7.58)	28.22 (6.92)	12.65 (1.29)
Soil nitrogen	High nitrogen	45.09 (2.49)	39.31 (2.07)	15.6 (1.46)
	Low nitrogen	46.02 (2.69)	39.27 (2.37)	14.71 (1.67)
Soil moisture	Control	42.47 (2.29)	38.04 (1.97)	19.49 (1.09)
	Drought	48.8 (2.78)	38.74 (2.11)	12.46 (1.25)

Data are expressed as means (SD).

### Relationship between CO_2_ diffusion and photosynthetic activity

**Fig. 7.**
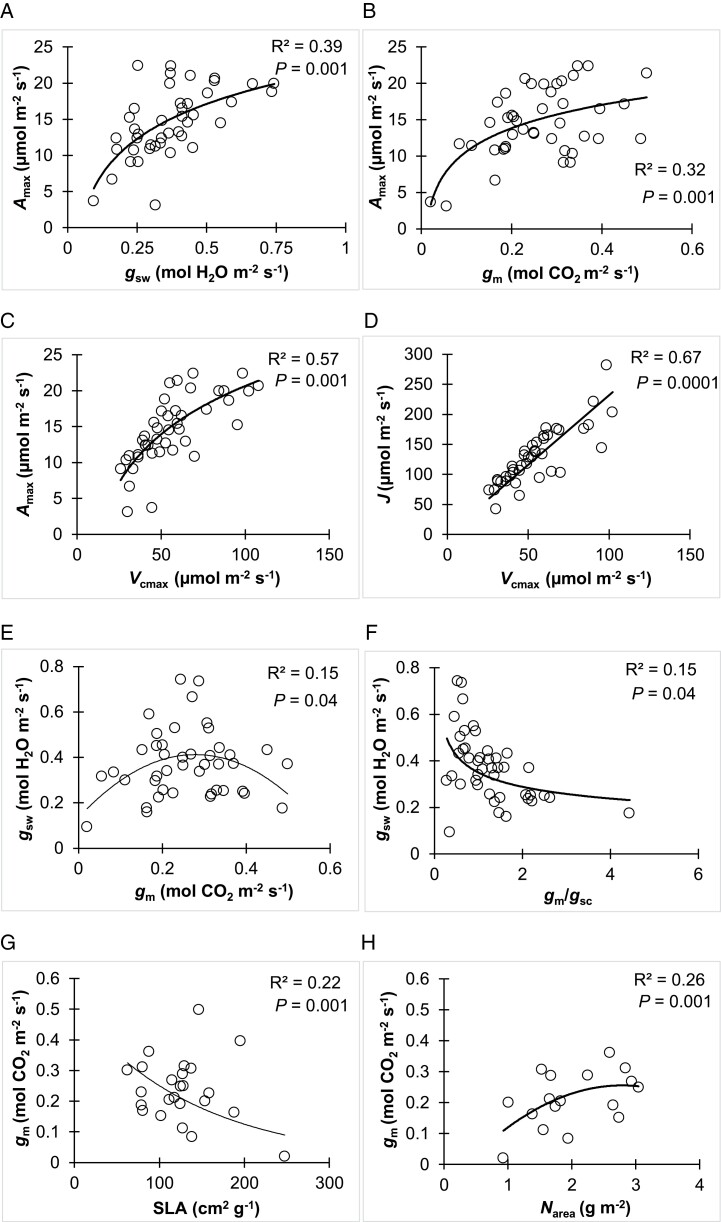
Relationship between light-saturated photosynthetic rate (*A*_max_), stomatal conductance (*g*_sw_), mesophyll conductance (*g*_m_), maximum rate of carboxylation (*V*_cmax_), electron transport rate (*J*), *g*_m_/*g*_sc_ ratio, specific leaf area (SLA) and per area leaf nitrogen concentration (*N*_area_). For *g*_m_/*g*_sc_ ratio, *g*_sw_ for water (mol H_2_O m^−2^ s^−1^) was divided by 1.6 to obtain *g*_sc_ (mol CO_2_ m^−2^ s^−1^).

*A*_max_ was strongly correlated to both *g*_sw_ and *g*_m_ (*P*=0.001) and to *V*_cmax_ (*P*=0.001) over all the studies (Fig. 7A–C). Based on the collected data, *g*_m_ was significantly correlated to *g*_sw_ (*P*=0.04). However, the relationship was not linear. *g*_m_ was the highest (0.4–0.5 mmol m^−2^ s^−1^) when *g*_sw_ values were intermediate (0.2–0.4 mol m^−2^ s^−1^), and lowest at high *g*_sw_ values (Fig. 7E).

We found a significant negative exponential relationship between SLA and *g*_m_ (*P*=0.001) (Fig. 7G) based on the collected data from studies that measured SLA (*n*=12). Leaf nitrogen content reported by three studies showed a significant correlation between *g*_m_ and N content per area (*N*_area_) (Fig. 7F). *g*_m_ increased with *N*_area_ until a saturation point (~0.25 mol m^−2^ s^−1^).

## Discussion

### Canopy level

The scaling up of photosynthesis from leaves to the canopy and stands (using the model of Farquhar *et al.* (1980)) requires a deep understanding of within-canopy variations in leaf morpho-physiology and the main drivers of foliage acclimation to the dynamic gradient of environmental conditions (light, temperature, vapor pressure deficit (VPD) and soil moisture) (Niinemets *et al.*, 2006; Buckley and Warren, 2014; Niinemets *et al.*, 2015). Unfortunately, pieces of knowledge regarding the variation of *g*_m_ within the canopy and its mechanistic basis are scarce, in particular for *Populus* spp. This situation may explain why most global carbon cycle models remain ‘*g*_m_-lacking’, with possible consequences, such as overestimation of the fertilization effect of CO_2_ on global gross primary production and underestimation of water-use efficiency (WUE) and canopy gross photosynthesis under future climate (Sun *et al.*, 2014b; Knauer *et al.*, 2019).

The steep and parallel increase of *g*_m_, *A*_max_, and *V*_cmax_ from the bottom to the top of the canopy found here for *Populus* spp. is in agreement with the findings of Niinemets *et al.* (2006) for *Quercus ilex* L., Montpied *et al.* (2009) for *Fagus sylvatica* L., and Warren *et al.* (2003) for *Pseudotsuga menziesii* (Mirbel) Franco. A decrease of *g*_m_ from the bottom to the top of the canopy was also reported (Bögelein *et al.*, 2012; Cano *et al.*, 2013). We observed a greater *g*_m_ limitation under shade conditions (mid and bottom of canopy), which may lead to an overestimation of canopy photosynthesis. Overall, our results highlight the need to incorporate the acclimation of *g*_m_ to light conditions along the canopy in process-based models.

We observed a significant inverse relationship between *g*_m_ and SLA, comparable to previous studies (Niinemets *et al.*, 2006; Montpied *et al.*, 2009; Tosens *et al.*, 2012*b*). This suggests that the increase in leaf thickness (lower SLA), e.g. in developing leaves and in leaves grown under higher light, may be associated with increased *g*_m_ (Tosens *et al.*, 2012*b*). Contrary to this, a positive relationship between *g*_m_ and SLA was demonstrated across *Solanum* species (Muir *et al.*, 2014), reflecting the effect of increased leaf density and mesophyll cell wall thickness on *g*_m_. These lines of evidence collectively demonstrate the complex nature of the relationship between SLA and *g*_m_, reflecting the circumstance that SLA is an inverse of the product of leaf thickness and density, which can respond differently to environmental drivers (Niinemets, 1999; Poorter *et al.*, 2009). The profile of *g*_m_ within the canopy observed here may be partially attributable to the morphological acclimation of *Populus* spp. foliage to light availability within the canopy. Moreover, this inverse relationship between SLA and *g*_m_ was used as an empirical model to estimate a maximum attainable *g*_m_ at different canopy layers for C_3_ plants and was implemented in the Community Land Model (CLM.4.5) (Sun *et al.*, 2014b; Knauer *et al.*, 2019).

The change in morphological traits and their role in the acclimation of *g*_m_ to a vertical gradient of environmental conditions within the canopy need additional investigation. For instance, shade acclimation of leaf morphology is associated with a lower surface area of chloroplasts exposed to intercellular air spaces (*S*_c_/*S*) and thicker chloroplasts (Hanba *et al.*, 2002; Niinemets *et al.*, 2006; Tosens *et al.*, 2012*b*; Peguero-Pina *et al.*, 2015). Species-specific leaf development patterns (i.e. evergreen sclerophyllous versus deciduous broadleaves) affect limitations to gas diffusion, thus determining the carbon balance of leaves (Marchi *et al.*, 2007). However, light acclimation may be species-specific and altered by water, soil nitrogen, and leaf ontogeny (Niinemets *et al.*, 2006; Tazoe *et al.*, 2009; Peguero-Pina *et al.*, 2015; Shrestha *et al.*, 2018). It is still unclear whether the *g*_m_ profile within the canopy is the result of the change in SLA.

Our results showing higher *g*_sw_ and *g*_m_/*g*_sc_ at the top of the canopy are in disagreement with the findings of Montpied *et al.* (2009) and Bögelein *et al.* (2012), suggesting a species- and environment-specific gradient of *g*_m_/*g*_s_. Temperature and VPD responses of *g*_m_ and *g*_s_ are different (Cano *et al.*, 2013), resulting in different diurnal patterns of *g*_m_ and *g*_s_. Then, the gradient of *g*_m_/*g*_s_ ratio along the canopy may drive the WUE at the canopy level and the midday depression of photosynthetic rate regardless of the level of isohydry of clones (Cano *et al.*, 2013; Buckley and Warren, 2014; Stangl *et al.*, 2019).

### Atmospheric CO_2_

The response of photosynthetic capacity and diffusion of CO_2_ to free-air CO_2_ enrichment considerably differed between species and experimental set-ups. The decrease in *A*_max_ and *g*_sw_ in response to elevated CO_2_ shown in our meta-analysis is in agreement with numerous studies on *Populus* spp. and other species (Ainsworth and Rogers, 2007; Medlyn *et al.*, 2013; DaMatta *et al.*, 2016), but is in disagreement with the findings of some other studies, e.g. Sigurdsson *et al.* (2001) and Uddling *et al.* (2009). For *g*_m_, the effect of growth CO_2_ changed among studies and some species having an intrinsic low *g*_m_ are more likely to respond to elevated CO_2_ than species with high intrinsic *g*_m_ (Niinemets *et al.*, 2011). However, several studies have reported that *g*_m_ may decrease or be unresponsive to CO_2_ enrichment (Singsaas *et al.*, 2004; Zhu *et al.*, 2012; Kitao *et al.*, 2015; Mizokami *et al.*, 2019). This suggests that the increase of *A*_max_ under elevated CO_2_ cannot be attributed solely to *g*_m_ variation (Singsaas *et al.*, 2004). The absence of *g*_m_ response to elevated CO_2_ complicates the research on mechanisms underlying this variation. Unlike *g*_m_, researchers have proposed some hypotheses such as least-cost theory, nitrogen limitation, and resources investment to explain the decrease of *A*_max_, *V*_cmax_, and *g*_s_ under elevated CO_2_ (Leakey *et al.*, 2009; Smith and Keenan, 2020).

### Copper stress

Similar to our findings, *g*_m_ remained unchanged in the herbaceous plant *Silene paradoxa* L., exposed to high Cu concentration, although *g*_s_ decreased significantly (Bazihizina *et al.*, 2015). In other cases of exposure to other heavy metals, like nickel (Ni), Velikova *et al.* (2011) reported a significant decrease in chloroplast CO_2_ content and mesophyll conductance in black poplar (*P. nigra* L.) exposed to 200 µM Ni under a hydroponic set-up (compared with control of 30 µM Ni). This reduction of *g*_m_ might be attributed to an alteration of leaf structure by toxic effects of high concentrations of heavy metals in mesophyll cells (Velikova *et al.*, 2011). Hermle *et al.* (2007), reported an acceleration of senescence and necrosis of mesophyll cells in *P. tremula* L. leaves exposed to Cu, Zn, Cd, and Pb at 640, 3000, 10, and 90 mg kg^−1^ soil, respectively, and a decrease of chloroplast size from the early stages of exposure. The study of Hermle *et al.* (2007) also reported the thickening of cell walls and change of their chemical composition in damaged mesophyll cells, which might have affected permeability of cell walls and diffusion of CO_2_ through them. Mercury (Hg; HgCl_2_ form) altered CO_2_ diffusion through aquaporins, a membrane channel of CO_2_ diffusion, in faba bean (*Vicia faba* L.) (Terashima and Ono, 2002) and significantly reduced *g*_m_ in *P. trichocarpa* Torr. & Gray. HgCl_2_ may also decrease *g*_m_ indirectly by disrupting carbonic anhydrase activity, as reported by Momayyezi and Guy (2018), who demonstrated that carbonic anhydrase activity is strongly associated with *g*_m_ variation in *P. trichocarpa* Torr. & Gray (Momayyezi and Guy, 2017).

### Soil nitrogen

The increase of *A*_max_ by the enhancement of *V*_cmax_ in response to more available soil nitrogen has been established in the literature. However, the possible contribution of *g*_m_ to this augmentation remains unexplored for several species. Our results showed a concomitant increase of *g*_m_ with a higher supply of N. A positive correlation between the level of expression of aquaporin genes (plasma membrane intrinsic proteins and tonoplast intrinsic proteins) and *g*_m_ has been reported (Hanba *et al.*, 2004; Flexas *et al.*, 2006; Kaldenhoff *et al.*, 2008; Perez-Martin *et al.*, 2014), although it is still unclear whether this is a direct effect or a pleiotropic effect reflecting simultaneous increase in *A*_max_, *g*_m_, and *g*_s_ (Flexas *et al.*, 2012). Recent studies have demonstrated that an increase in *g*_m_ has coincided with an increase in the amount of aquaporins after fertilization (Miyazawa *et al.*, 2008b; Zhu *et al.*, 2020). The biochemical limitation to photosynthesis was relatively low (16%) and the absolute contribution of this limitation to the decrease in *A*_max_ under low nitrogen was much lower again (1.5%). This suggests that the limitations to photosynthesis resulting from low soil nitrogen are more attributable to CO_2_ diffusion for *Populus* spp.

### Soil moisture

Although many studies showed a decline of *g*_m_ in response to soil water deficit (Flexas *et al.*, 2009; Galle *et al.*, 2009; Tosens *et al.*, 2012*a*), it remains unclear if this limitation is happening within the mesophyll environment or occurs as a result of a stomatal limitation, which decreases intercellular CO_2_ (*C*_i_). Ma *et al.* (2021) reported that, across a broad range of species, *g*_m_ and *g*_s_ decline concomitantly, which has the effect of keeping the *g*_m_/*g*_sc_ ratio constant for all species and between well-watered and water-stressed plants, but with variation between plant functional types. We report similar *g*_m_/*g*_sc_ ratios within our soil moisture dataset. However, reports in poplar have shown that this concomitant decline is not present all the time or within the full range of *g*_m_ and *g*_s_ values observed. Théroux Rancourt *et al.* (2015) showed that, in hybrid poplar, *g*_m_ remained unchanged (~0.3 mol m^–2^ s^–1^) following soil drying until Ψ _leaf_≈−1.2 MPa, after which *g*_m_ decreased significantly. Our results showed that although *g*_m_/*g*_s_ increased under water deficit conditions, stomatal conductance was, in absolute term, the most important limitation to *A*_max_, as reported elsewhere (Cano *et al.*, 2013). In a trial on *Quercus robur* L. and *Fraxinus angustifolia* Vahl grown in the field, Grassi and Magnani (2005) reported a concomitant decrease of both *g*_s_ and *g*_m_ in a dry year (Ψ _soil_≈−1.7 MPa), compared with a wetter year (Ψ _soil_≈−0.2 MPa). In *P. tremula* L., *g*_m_ significantly declined when Ψ _leaf_ of saplings dropped from −0.3 to −0.7 MPa due to applied osmotic stress (Tosens *et al.*, 2012*a*). Simultaneously, drought stress induced a decrease in SLA accompanied with an increase in the cell wall thickness and a decrease in the chloroplast surface area exposed to the intercellular air space per unit leaf area (Tosens *et al.*, 2012*a*). Other studies have shown that biochemical changes induced by drought stress could decrease CO_2_ diffusion to carboxylation sites in the chloroplast (Miyazawa *et al.*, 2008a).

Adaptation to the local environment might be a key driver of *g*_m_ variation among taxa, similarly to other morpho-physiological traits. Interspecific and intraspecific differences in *g*_m_ from mesic versus xeric environments (*Quercus* spp. and *Eucalyptus* spp.) were reported by Zhou *et al.* (2014). Their study showed that *g*_m_, as well as *g*_s_, *V*_cmax_, and *J* of species from drier regions was less sensitive to water deficit, which maintains transpiration and photosynthesis activity at higher rates under drought, compared with species from the mesic environment. Marchi *et al.* (2008) observed that structural protection of mesophyll cells had a priority over functional efficiency of photochemical mechanisms in *Olea europaea* L. (evergreen sclerophyllous) but not in *Prunus persica* L. (deciduous broadleaf), depending on age-related variation in mesophyll anatomy.

## Conclusion and future directions

The present review shows that *g*_m_ in *Populus* spp. varies predictably along light gradients and that it responds to changes in soil moisture and nutrient availability, but is not affected by metal concentration and increasing atmospheric CO_2_ concentration. Although metabolic processes noticeably influence the response of *g*_m_ to environmental changes, physical constraints through leaf development and ageing need to be considered in scaling photosynthesis from leaf to canopy, and in breeding programs for high WUE. Because fast-growing *Populus* spp. trees are important players in combating climate change, mitigating carbon emissions to some extent, comparisons of genotypes with different adaptations to changing environments and breeding for novel genotype–climate associations are urgently needed. This study shows that the variability of *g*_m_ in different experimental conditions offers a potential indicator for improving *Populus* spp. productivity and resilience. However, more research is yet needed, also combined with anatomical studies, to better understand the sources of variation of CO_2_ diffusion through the mesophyll and their consequences on carbon assimilation and growth.

Moreover, determination of the efficiency and optimal age for early selection of fast-growing poplar clones require an understanding of the genetic control and age-based genetic correlations for traits related to *g*_m_ and growth. For that, a detailed evaluation of the genotypic control of the variances and clonal heritability of *g*_m_ is needed. Finally, the identification of molecular bases of the regulation of *g*_m_ is necessary to further refine a multi-criteria early selection approach of poplar clones dedicated to the future forestry capable of ensuring better productivity and increased resistance to environmental stresses (frost, drought, water logging, heavy metals, heat waves, etc.).

## Supplementary data

The following supplementary data are available at *JXB* online.

Table S1. Analysis of variance of the effect of different factors on photosynthesis-related traits.

erab127_suppl_Supplementary_Table_S1

## Data Availability

Soil moisture data from Théroux-Rancourt are available at Dryad Digital Repository (https://doi.org/10.5061/dryad.7sqv9s4s0); Benomar data are available at Dryad Digital Repository (https://doi.org/10.5061/dryad.9cnp5hqhp); Tognetti data are available at Dryad Digital Repository (https://doi.org/10.5061/dryad.w3r2280qq). All other datasets generated for this study are available from the corresponding author upon request.
