# Supplementary material for: A meta-analysis of mesophyll conductance to CO2 in relation to major abiotic stresses in poplar species
Source: J Exp Bot. 2021 Mar 19;72(12):4384–400. doi: 10.1093/jxb/erab127 (PMC8163042; doi:10.1093/jxb/erab127)
Supplement: erab127_suppl_Supplementary_Table_S1 [file erab127_suppl_supplementary_table_s1.pdf]

Table S1. Analysis of variance of the effect of different factors on photosynthetic-related traits

		A_{max}		g_{sw}		g_m		g_m/g_{sc}	
Source	DF	F	P-value	F	P-value	F	P-value	F	P-value
Canopy level	2	96.65	**<0.001**	37.25	**<0.001**	50.68	**<0.001**	4.59	**0.001**
Ambient CO_2	1	0.63	0.43	1.33	**0.25**	1.33	0.25	0.37	0.34
Copper stress	2	0.26	0.77	3.95	**0.032**	0.21	0.81	0.04	0.95
Soil nitrogen	1	32.30	**<0.001**	5.29	**0.024**	8.74	**0.004**	1.78	0.24
Soil moisture	1	4.99	**0.029**	17.20	**<0.001**	12.18	**0.001**	**4.28**	**0.044**

A_{max}: light-saturated photosynthetic rate; g_{sw}: stomatal conductance; g_m : mesophyll conductance. In g_m/g_{sc} ratio, g_{sw} for water (mol H_2O m^{-2} s^{-1}) was divided by 1.6 to obtain g_{sc} (mol CO_2 m^{-2} s^{-1}).
